# Development and evaluation of a self-generated foam–resin composite sand consolidation system for offshore oil reservoirs

**DOI:** 10.1371/journal.pone.0338643

**Published:** 2025-12-05

**Authors:** Mengsheng Jiang, Shanfa Tang

**Affiliations:** 1 School of Petroleum Engineering, Yangtze University, Wuhan, China; 2 Hubei Key Laboratory of Oil and Gas Drilling and Production Engineering (Yangtze University), Wuhan, Hubei, China; 3 School of Petroleum Engineering, Yangtze University: National Engineering Research Center for Oil and Gas Drilling and Completion Technology, Wuhan, China; 4 State Key Laboratory of Low Carbon Catalysis and Carbon Dioxide Utilization, Wuhan, China; China University of Petroleum Beijing, CHINA

## Abstract

Sand production poses a major challenge in offshore heavy oil development, leading to casing erosion, equipment damage, and wellbore instability, which ultimately results in production decline. Conventional mechanical and chemical sand control methods often exhibit limited effectiveness in fine-grained or high-salinity formations and may induce formation damage or high operational costs. This study developed a self-generated foam–resin composite sand consolidation system to address these issues. The system integrated a controllable gas-generating subsystem and a melamine–formaldehyde (MF) resin matrix, thus enabling in-situ foam generation and sand consolidation. Laboratory experiments were conducted in three stages: optimization of gas and foaming agent subsystems, formulation and evaluation of the resin-based consolidant, and integration testing of the composite system. The optimized formulation resulted in controllable gas production, high foam stability, and consolidated sand cores with compressive strength >7 MPa. The system exhibited thermal stability up to 90 °C, salinity resistance, moderate permeability (~4 μm^2^), non-adhesion to metal surfaces, and negligible sand production (<0.1%) under dynamic water flooding. These results indicate that the foam–resin composite system provides an efficient, environmentally compatible, and cost-effective solution for sand control in complex offshore heavy oil reservoirs.

## 1. Introduction

After decades of production, the water cut in Chinese offshore oilfields has increased. In particular, the liquid production water cut of Bohai Oilfield has exceeded 80% [1], indicating that it has entered development stage. To maintain oil production, high liquid production are currently adopted for production [2]. Consequently, sand production has become increasingly severe with increasing water cut.

Reservoir sand production is a common and highly hazardous issue in oil and gas development, and its impacts are mainly reflected in three aspects. First, hard sand particles enter the wellbore and surface systems along with fluids, causing severe abrasive damage abrasive wear to downhole equipment such as casings and oil production tubing [3,4]. This results in increased equipment wear, shortened service life, and increased maintenance costs. Second, when numerous sand particles enter the wellbore and accumulate gradually, they not only can cause wellbore blockage and reduce production [5,6], but also may damage the wellbore structure when sand production exceeds a certain threshold, leading to oil well shutdown or even abandonment. Third, under high flow rate conditions, the scouring effect of fluids on the rock framework is enhanced, causing the reservoir framework to gradually become unstable [7]. This results in the collapse of pore structures and damage to reservoir integrity, further exacerbating the sand production problem and reducing the development potential of the reservoir [8,9].

Sand consolidation technologies for oil and gas wells mainly include three categories: mechanical sand consolidation, chemical sand consolidation, and composite sand consolidation. Represented by sand consolidation pipe strings and gravel packing, mechanical sand consolidation has advantages such as simple construction, wide adaptability, and the ability to extend the production cycle. However, its effectiveness is limited in fine silt layers and high-pressure formations, and it is prone to sand blockage [10]. Chemical sand consolidation is widely used in oilfields, with advantages including no occupation of wellbore space, simple construction, and suitability for multi-layer and fine silt reservoirs [11,12]. Common methods include resin-coated sand and resin sand consolidation agents. The currently studied systems mainly include epoxy, phenolic, urea-formaldehyde, furan, and melamine-formaldehyde resins (MF resins) [13]. Epoxy resins are widely used due to their high bonding strength [14–16]; phenolic resins exhibit excellent temperature resistance [17,18]; urea-formaldehyde resins (UF resins) are low-cost and suitable for low-temperature reservoirs [19]; furan resins have good stability under high-temperature conditions [20,21]; and melamine-formaldehyde resins possess excellent water resistance and mechanical properties [22,23]. Numerous experiments and field applications have shown that different systems can achieve good sand consolidation effects under specific formation conditions. Combining the advantages of mechanical and chemical methods, composite sand consolidation can form multiple protective barriers in the wellbore, and is particularly suitable for high-yield fine siltstone reservoirs [24,25].

Despite certain progress, existing sand consolidation technologies still have shortcomings. Mechanical sand consolidation has poor adaptability to fine silt and high-pressure reservoirs, and post-treatment is difficult [26]. Chemical sand consolidation agents have insufficient stability in high-temperature and high-salinity environments; although some systems have high consolidation strength, they cause significant damage to reservoir permeability [27]. Additionally, they generally have issues such as high environmental sensitivity and high construction costs. While composite sand consolidation improves the limitations of single methods, it has complex processes and increased costs, and its long-term stability and large-scale promotion effects remain to be verified.

Therefore, how to achieve efficient, stable, economical, and environmentally friendly sand consolidation in complex formation environments remains a key scientific and engineering problem that needs to be urgently solved. However, few studies have combined in-situ gas generation with resin consolidation to realize foam-assisted sand stabilization under reservoir conditions. Focusing on offshore heavy oil fields, Therefore, this study aims to develop a self-generated foam–resin composite system that combines controllable gas generation, stable foam, and enhanced resin consolidation to overcome the limitations of existing methods. Compared with traditional foam–resin or gas-assisted consolidation methods, the proposed self-generated foam–resin system removes the need for external gas injection and complex on-site operations. Its in-situ foaming mechanism allows more controllable foam generation and placement, improving consolidation uniformity and reducing formation damage under high-temperature, high-salinity conditions.

## 2. Methods and experiment

The self-generated foam–resin composite sand consolidation system consists of two primary components: a self-generated foam system and a resin-based sand consolidation system. To systematically develop and evaluate this in situ foam–resin composite sand consolidation technology, the experimental work was conducted in three consecutive stages. In the first stage, the self-generated foam system was developed and optimized by investigating the interaction between the gas-generating agents and the foaming agent. In the second stage, various resin types were screened, and the key formulation parameters were optimized to establish a high-performance resin-based sand consolidation system. In the third stage, the two subsystems were integrated, and the composite system was comprehensively evaluated in terms of compatibility, sand consolidation performance under simulated reservoir conditions, and the underlying mechanisms contributing to enhanced sand consolidation. The materials and detailed experimental procedures for each stage are described in the following sections.

### 2.1. Materials

The main experimental materials required for this study are listed in Table 1.

**Table 1:** Main experimental materials for this study.

Reagent	Manufacturer
NaNO_2_	Merck(Germany)
NH_4_Cl	Merck(Germany)
KH-550	Nanjing Capatue Chemical
20201	Shandong Baomo
20202	Shandong Baomo
20203	Huading Hongji
20204	Beijing Hengju
QP-2	Self-Developed
MF Resins	Merck(Germany)
UF Resins	Merck(Germany)

### 2.2. Equipment

The main experimental equipments required for this study are listed in Table 2.

**Table 2:** Experimental equipments for this study.

Instrument Name	Manufacturer
Drum Flowmeter	Ritter (Germany)
Glass Sand-Packed Tube (Fig 1a)	Self-Designed and Assembled
Steel Sand-Packed Tube (Fig 1b)	Self-Designed and Assembled
Water-Based Core Permeability Measuring Device	Jiangsu Hua’an Scientific Research Instruments Co., Ltd.
Rock Mechanics Testing Equipment (Fig 1c) (Figs 3–7)	Self-Designed and Assembled
Interfacial Tensiometer	TA (USA)
Foam Scanner	Teclis Interface Technology (France)

**Fig 1:**
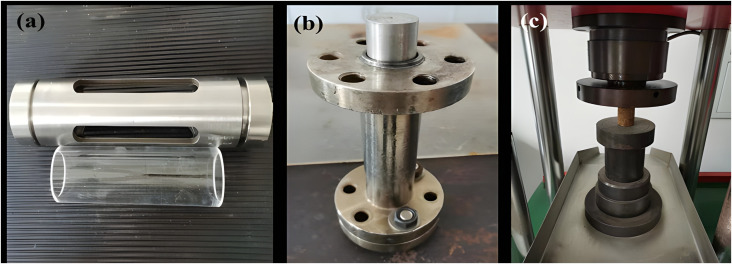
Custom-designed and assembled sand-filling tubes and rock mechanics testing equipment. (a) Glass sand-filling tube. (b) Steel sand-filling tube. (c) Rock mechanics testing equipment.

**Fig 2:**
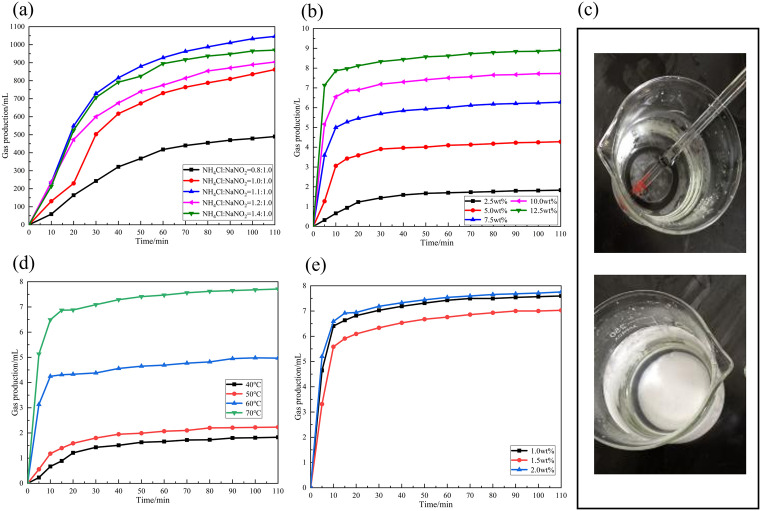
Effects of formulation parameters on the gas-generation behavior of the self-generated system. (a) Effect of Different Molar Ratios on Gas Production; (b) Effect of Different Concentrations of NaNO_2_ on Gas Production of the System; (c) Dissolution of NaNO_2_ at Different Concentrations; (d) Effect of Different Reaction Temperatures on Gas Production; (e) Effect of Different Catalyst Concentrations on Gas Volume.

**Fig 3:**
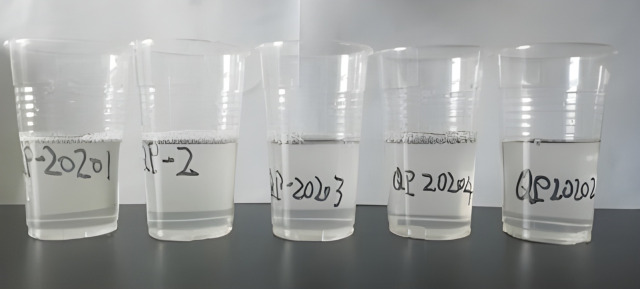
Compatibility between foaming agent and injection water.

**Fig 4:**
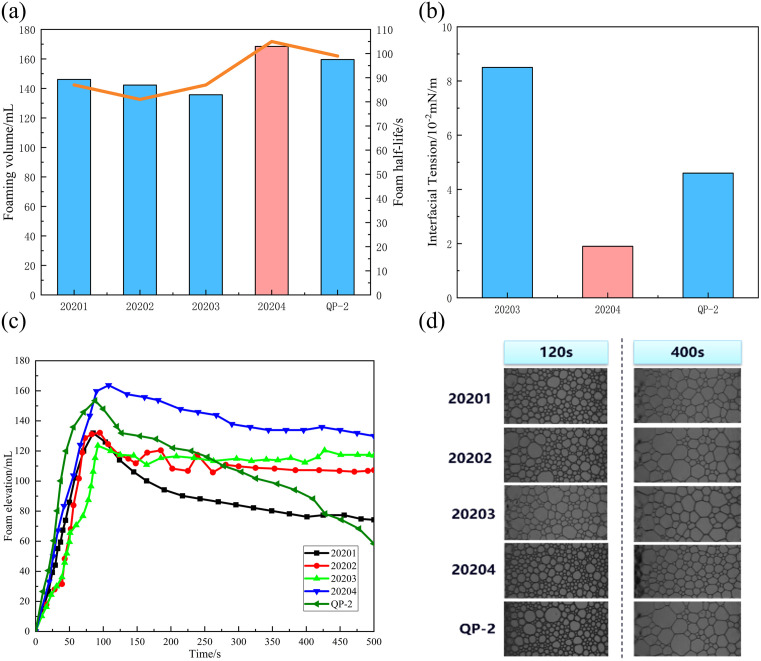
Comparative foam performance and morphological characteristics of different foaming agents. (a) comparison of foaming volume of different foaming agents; (b) comparison of interface performance of different foaming agents; (c) variation trend of foam height with time; (d) foam morphology at different time points.

**Fig 5:**
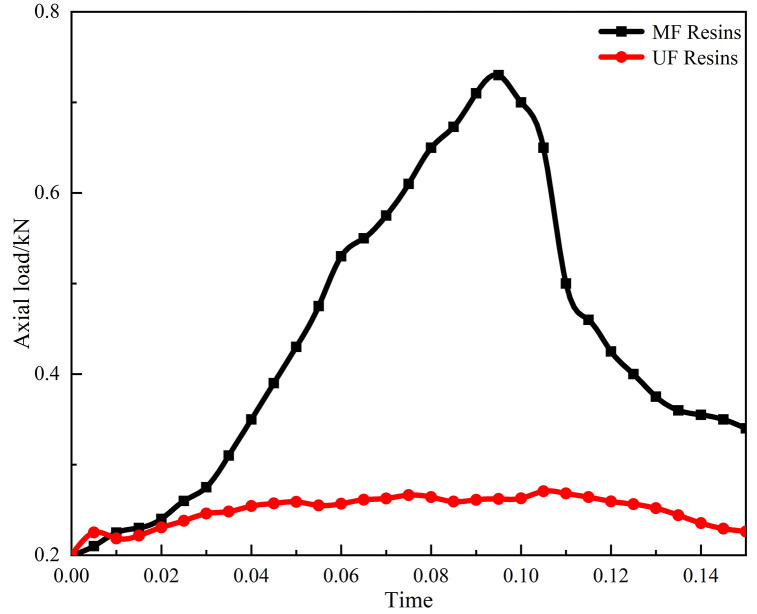
Compressive strength test curves of cores consolidated with two amino resins under the same conditions.

**Fig 6:**
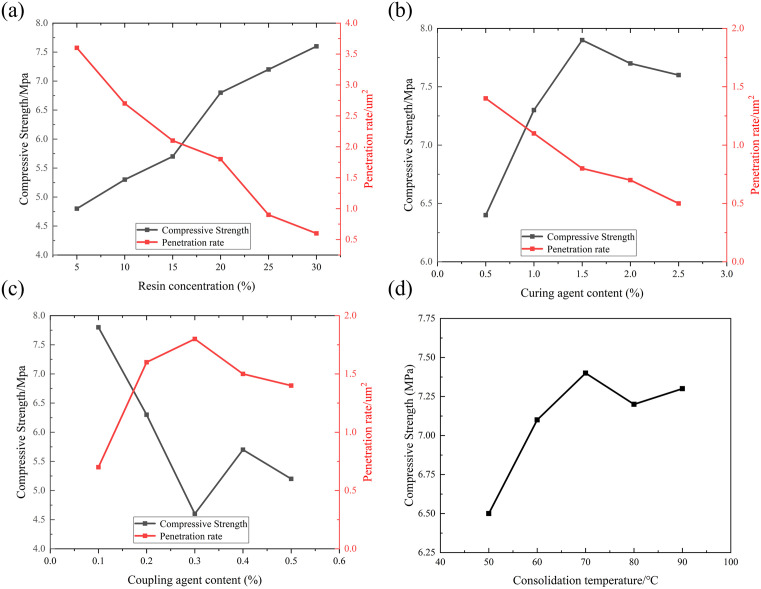
Effects of key formulation and processing parameters on sand consolidation performance: (a) MF Resin Concentration, (b) Curing Agent Concentration, (c) Coupling Agent Concentration, (d) Curing Temperature.

**Fig 7:**
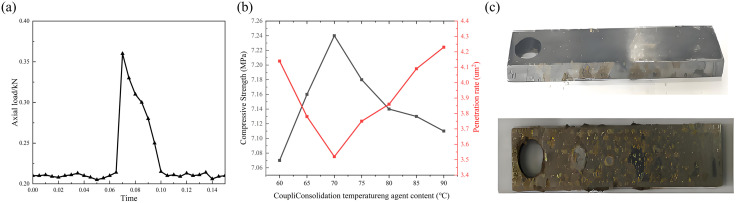
Performance evaluation of the autogenous foam composite sand consolidation system (a) compressive strength test curve of autogenous foam composite sand consolidation system; (b) evaluation of sand consolidation effect of autogenous foam composite sand consolidation system; (c) surface changes of steel sheet after experiment.

### 2.3. Methods

#### 2.3.1. Self-foaming system development.

A series of experiments were conducted to investigate the effects of main reagent ratio, main reagent concentration, reaction temperature, and catalyst concentration on gas generation behavior. All experiments followed this general procedure: First, main reagent solutions (with varying molar ratios of sodium nitrite to ammonium chloride or different concentrations) with a total volume of 150 mL and a specific volume of catalyst solution were prepared according to the experimental design. Then, 50 mL of the catalyst solution was transferred into a 500 mL three-neck flask, followed by the addition of the main reagent solution. The flask was connected to a gas circuit and a gas flow meter, and immersed in a water bath set to the target temperature. After temperature stabilization, data from the gas flow meter were recorded simultaneously with the initiation of reaction timing. The reaction was maintained until gas generation nearly ceased.

The specific experimental variables were as follows:

(1) Effect of Main Reagent Ratio: Solutions were prepared with varying molar ratios of NaNO₂ to NH₄Cl, while maintaining the water bath temperature at 70°C and a specified catalyst concentration [28].
(2) Effect of Main Reagent Concentration: Based on the determined optimal reagent ratio, main reagent solutions with different concentrations were prepared and tested [29].
(3) Effect of Reaction Temperature: Using the optimized main reagent concentration and ratio, the general procedure described above was repeated at water bath temperatures of 40°C, 50°C, 60°C, and 70°C [30,31].
(4) Effect of Catalyst Concentration: Experiments were conducted using catalyst solutions with concentrations of 1.0 wt%, 1.5 wt%, and 2.0 wt%, under the optimal main reagent conditions and a water bath temperature of 70°C.

#### 2.3.2. Screening and optimization of the foaming agent.

**Interfacial tension measurement:** Interfacial tension between oil and water was measured via Texas-500 tensiometer: inject aqueous solution into capillary to 1–2 mm below top (no bubbles), inject oil to form droplet via microsyringe then fill and seal capillary; mount capillary, adjust to experimental temperature, rotate at 8–12 ms/rev, locate and level oil column; record bottom (d₁) and top (d₂) readings to get diameter Y = d_1_–d_2_ (10^−4^ m, measure length if ＜4 × diameter), determine oil/water density and water refractive index, then calculate γ via formula [32,33].

For oil column length greater than four times its diameter:


$$\begin{array}{c} \gamma =\frac{\text{1}\mathrm{.2336}\Delta \rho {\left(\frac{\text{Y}}{\text{n}}\right)}^{\text{3}}}{P^{\text{2}}} \end{array}$$ (1)

For oil column length less than four times its diameter:


$$\begin{array}{c} \gamma =\frac{\text{1}.\text{2}\text{3}\text{3}\text{6}\Delta \rho \;\text{f}\left(\frac{\text{Z}}{\text{Y}}\right){\left(\frac{\text{Y}}{\text{n}}\right)}^{\text{3}}}{P^{\text{2}}} \end{array}$$ (2)

where γ is the oil–water interfacial tension (mN/m), Δρ is the density difference between oil and water (g/cm³), Y is the oil column diameter (10 ⁻ ⁴ m), P is the inverse of the rotation speed (ms/rev), n is the refractive index of the aqueous phase, and f(Z/Y) is a correction factor for short oil columns.

**Foam characterization and measurement of foaming properties:** Foam was generated in a foam micro-scanner, with a CCD camera recording formation and collapse processes, and software analyzing images to quantify parameters; 200 mL surfactant solutions of different concentrations were added to the test column, electrodes measured liquid volume-conductivity relationship and recorded initial volume V_L0_, nitrogen was injected at a controlled rate through a porous plate until preset time t0—foam volume VF0 was foaming ability, remaining liquid volume V_L1_ gave maximum liquid-holding capacity V_LMAX_ = V_L0_ − V_L1_, time t_1_ when liquid-holding capacity decreased to half VLMAX was recorded, and foam half-life t_3_ = t_1_ − t_0_.

#### 2.3.3. Research and development of resin-based sand solidification systems.

**Preparation of resin-filled sandstone core:** Preparation of resin-filled sandstone core: Cleaned quartz sand with controlled particle size was washed with distilled water, oven-dried at 65 °C, and packed in layers into glass tubes with stainless steel screens at both ends, tapped lightly for uniform density; after water saturation by displacement pump, 1−2 pore volumes of resin system were injected at constant flow rate, sealed and cured in an oven at 60 °C for 24 h, then taken out, cooled to room temperature and polished to make ends flat and perpendicular to core axis; mechanical strength was tested by uniaxial compression according to SY5276−91 standard, compressive strength was calculated from peak load and cross-sectional area after loading to failure; permeability was measured by core flooding experiment, core was clamped with confining pressure, water was injected at constant flow rate to steady state, and calculated via Darcy’s law K=(QμL⁄∆pA)×10^(−1) using flow rate, fluid viscosity, pressure difference and core geometric dimensions [34,35].

**Compressive strength measurement of resin-bonded sand Cores:** The compressive strength of resin-bonded sand cores was measured using rock mechanics testing equipment. The consolidated core was placed into the testing apparatus as illustrated in Figs 3–7. The instrument was operated to record the variation of applied load over time until core failure occurred. The compressive strength ZZZ of the consolidated core was calculated using the following equation:


$$\begin{array}{c} Z=\frac{F}{A}\times \text{0}.\text{0981} \end{array}$$ (3)

where Z is the compressive strength of the consolidated core (MPa), F is the load at failure (N), and A is the cross-sectional area of the core (cm²).

The permeability of consolidated cores was determined using a physical core-flooding experimental setup. The length and diameter of each core were accurately measured with a vernier caliper. The core was then placed into a core holder, and the front and rear ends were tightened to secure the core, ensuring proper sealing. The core holder was connected to the core-flooding apparatus, and the confining pressure was set to 2 MPa plus the injection pressure. Water was injected at a constant rate using a displacement pump, and the stabilized injection pressure was recorded. The water-phase permeability (K) of the consolidated core was calculated using Darcy’s law:


$$\begin{array}{c} K=\frac{Q\mu L}{\Delta pA}\times {\text{10}}^{-\text{1}} \end{array}$$ (4)

where K is the water-phase permeability (μm^2^), Q is the water flow rate (cm^3^·s⁻^1^), μ is the water viscosity (Pa·s), L is the core length (cm), ΔP is the pressure difference (Pa), and A is the cross-sectional area of the core (cm^2^).

#### 2.3.4. Self-generated foam–resin composite sand consolidation system.

The optimal formulation was determined based on preliminary optimization (12.5% NaNO₂, 15% NH₄Cl, 2.5% catalyst, 0.5% foaming agent 20204, 25% melamine-formaldehyde resin, 1% curing agent, 0.25% coupling agent KH-550, prepared with KL10–4 oilfield injection water), and its foaming, consolidation performance and mechanism were evaluated; 60–80 mesh quartz sand was packed into steel sand-filling tubes as Section 2.3.3, a 100-mesh steel screen and steel dummy core were placed at the sand column end, 15 MPa was applied by rock mechanics equipment to compact and simulate underground high-pressure environment, cured at 60–90°C for 24 h, and compressive strength and water-phase permeability were measured as Section 2.3.3; plug-free performance test: ① catalyst-free system was coated on clean steel sheet, ② the same system was coated on fine sand-covered steel sheet, both cured at 65°C for 24 h before surface observation; sand-loss evaluation: 40–60, 60–80, 80–100, 100–120 mesh quartz sand was packed into steel sand-filling tubes as Section 2.3.3, compacted at 15 MPa, cured at 60°C for 24 h, connected to core-flooding apparatus and flushed with injection water at 50 mL/min for 60 min, and sand-loss rate was calculated by measuring mass before and after flushing.

## 3. Results and discussion

### 3.1. Performance of self-foaming system

#### 3.1.1. Performance of autogenous gas systems.

The systematic investigation of reaction parameters revealed distinct optimization pathways for enhancing gas generation efficiency. As shown in Fig 2a, the molar ratio of NaNO₂ to NH₄Cl exerted a profound influence on cumulative gas production, with the maximum yield achieved at 1.0:1.1. This optimum corresponds to a stoichiometric balance that promotes the forward reaction through favorable ionic equilribrium, whereas excess NH₄Cl displaced the equilibrium reversibly, diminishing both reaction efficiency and total output. Complementarily, the concentration of NaNO₂ significantly modulated reaction kinetics and gas release characteristics (Fig 2b). Increasing its concentration from 2.5 wt% to 12.5 wt% enhanced the initial gas release rate and cumulative volume, reaching approximately 7 L within 5 min at the highest concentration. Nevertheless, supersaturated conditions induced salt precipitation and crystallization during evaporation (Fig 2c), underscoring the need to balance performance with physicochemical stability in field applications [36].

Temperature was identified as a critical accelerator of the gas-forming reaction, with Fig 2d illustrating a marked increase in both rate and yield above 60°C. This threshold reflects the thermally activated nature of the reaction pathway, confirming the necessity of maintaining reservoir-compatible temperatures for optimal performance. Concurrently, catalyst concentration governed the initial kinetics, as higher dosages up to 1.5 wt% considerably shortened the induction period and intensified early-stage gas release (Fig 2e). Beyond this level, a plateau in gas production emerged, indicative of a self-sustaining mechanism mediated by reactive intermediates. It is noteworthy that excessive catalyst under acidic conditions risks promoting the decomposition of NO₂⁻ into toxic nitrogen oxides, necessitating careful dosage control to maximize efficiency while mitigating safety hazards.

#### 3.1.2. Evaluation of foaming agent performance.

Compatibility and foaming performance of five candidate surfactants were systematically evaluated under simulated reservoir conditions. All agents at 5000 mg/L demonstrated excellent compatibility with filtered KL10−4 injection water, showing no phase separation or precipitation after 48 h of aging at reservoir temperature (Fig 3). Foaming capacity and stability were quantitatively assessed using complementary techniques—the Waring blender method and foam scanner. Notably, foaming agent 20204 exhibited superior performance, achieving a foam volume of 410 mL and a liquid half-life of 442 s, which corresponds to a foam stability index of 135,915 mL·s. This value surpasses those of other candidates by an order of magnitude, underscoring its exceptional foam-forming and stabilization capabilities under reservoir-relevant conditions [37]. The consistent trends observed between the two evaluation methods validate the reliability of the screening protocol and establish foaming agent 20204 as the optimal choice for further application (Table 3).

**Table 3:** Comparison of foam performance among different foaming agents.

No.	Interfacial Tension mN/m	Foam Scan Method			Waring Blender Stirring Method		
		Foaming Volume/mL	Drainage Half-Life/s	Comprehensive Foam Value/mL·s	Foaming Volume/mL	Drainage Half-Life/s	Comprehensive Foam Value/mL·s
20201	4.5 × 10^−1^	146.1	87	9533	320	262	62880
20202	9.8 × 10^−1^	142.3	81	8644.7	240	33	5940
20203	8.5 × 10^−2^	135.7	87	8854.4	260	111	21645
20204	1.9 × 10^−2^	168.5	105	13269.4	410	442	135915
QP-2	4.6 × 10^−2^	159.6	99	11850.3	380	408	116280

A comprehensive evaluation of foaming performance and interfacial properties confirmed the superior efficacy of foaming agent 20204. As illustrated in Fig 4a, it generated the largest initial foam volume, a result consistent with its exceptional interfacial activity. Interfacial tension measurements (Fig 4b) further revealed that three agents reduced oil–water tension to the order of 10⁻^2^ mN/m, with 20204 achieving the lowest value, followed by QP-2 and 20203. This ultra-low interfacial tension promotes enhanced surfactant adsorption at the gas–liquid interface, contributing to improved foam stability [38]. Temporal evolution of foam height (Fig 4c) demonstrated that 20204 exhibited minimal decay over 500 s, indicating remarkable resistance to coalescence and liquid drainage. While all foams underwent progressive liquid loss, 20204 retained approximately 5% liquid content at 400 s—a point at which other systems were nearly depleted—reflecting its superior foam strength and structural integrity. Microscopic analysis (Fig 4d) corroborated these findings, showing that 20204 maintained uniform bubble size distribution with minimal coarsening over time, in contrast to significant structural degradation observed in other foams [39]. The integration of multiparameter assessments—encompassing compatibility, foamability, interfacial behavior, drainage dynamics, and morphological evolution—collectively establishes 20204 as the optimal foaming agent for the self-generated foam–resin sand consolidation system. Its ability to deliver high foam volume, extended stability, and microstructural homogeneity ensures the formation of a stable gas phase, thereby enabling effective coupling with the MF-resin subsystem under reservoir conditions.

### 3.2. Performance of resin sand consolidation system

#### 3.2.1. Resin type screening and performance comparison.

Tests on the compatibility of different resin types with foaming agent 20204 and their influence on foam properties (Table 4) showed that melamine-formaldehyde resin (MF Resins) and urea-formaldehyde resin (UF Resins) generated relatively high foam volumes of 132 mL and 135 mL, respectively, at the same concentration, while phenolic resin and modified furan resin produced only 85 mL and 96 mL of foam. In terms of foam stability, UF resin achieved the highest half-life (60 min) and comprehensive foam value (6075 mL·min^-1)^, followed by MF resin (35 min, 3465 mL·min^-1^), indicating good compatibility with the surfactant system.

**Table 4:** Foaming effect of different types of resins.

Resin Type	MF Resins	phenolic resin	modified furan resin	UF Resins
Foaming Volume, mL	132	85	96	135
Half-Life, min	35	18	20	60
Comprehensive Foam Value, mL·min^-1^	3465	1147.5	1440	6075

However, as shown in Fig 5, UF resin exhibited poor consolidation strength in sand pack tests, whereas cores consolidated with MF resin showed significantly higher compressive strength. Considering both foam stability and consolidation strength, MF resin was identified as the optimal choice for the self-generated foam–resin composite system.

#### 3.2.2. Effect of MF resin concentration on foaming and consolidation performance.

As shown in Table 5, when the MF resin concentration increased from 5% to 30%, the foam volume slightly decreased (from 373 mL to 326 mL), but the half-life significantly extended from 472 s to 675 s. The intrinsic viscosity of the resin delayed liquid drainage, thereby enhancing foam stability. The comprehensive foam value increased accordingly to 165,037.5 mL·min^-1^, confirming that higher resin concentrations prolong foam longevity through a stabilizing effect [40].

**Table 5:** Results of foaming performance of MF resin with different concentrations.

Content,%	5	10	15	20	25	30
Foaming Volume, mL	373	362	367	351	343	326
Half-Life, min	472	493	491	564	627	675
Comprehensive Foam Value, mL·min^-1^	132042	133849.5	135147.8	148473	161295.8	165037.5

Fig 6a illustrates the effect of resin concentration on consolidation performance: as the concentration increased, the compressive strength of the cores continuously improved, but permeability gradually decreased. This is because higher resin content strengthens sand grain bonding while simultaneously blocking more pore channels. Although high concentrations enhance strength, they excessively reduce permeability, potentially impairing formation fluid flow. Balancing strength, permeability, and cost, a resin concentration of 25% was determined as the optimal value [41].

#### 3.2.3. Optimization of curing agent concentration.

As shown in Fig 6b, when the curing agent concentration increased from 0.5% to 2.5%, the compressive strength of the cores initially increased and then stabilized. Beyond 1.5%, no further improvement in polymerization was observed, as the condensation reaction was complete. Excess curing agent occupied pore space, leading to continued permeability reduction. Therefore, a curing agent concentration of 1% was selected to ensure adequate strength while minimizing permeability damage.

#### 3.2.4. Interfacial enhancement by coupling agent KH-550.

The addition of silane coupling agent KH-550 improved the interfacial bonding between the resin and quartz sand. Fig 6c shows that as the KH-550 concentration increased, the compressive strength of the cores first increased and then decreased, peaking at 0.25%. Permeability showed an opposite trend, decreasing sharply initially and slightly recovering at higher concentrations. An appropriate amount of KH-550 enhances interfacial connection through siloxane bond formation, whereas excessive addition may weaken hydrogen bonding and reduce consolidation effectiveness. Considering both technical and economic factors, 0.25% was selected as the optimal concentration [42].

#### 3.2.5. Effect of temperature on consolidation behavior.

After curing 60–80 mesh quartz sand samples at 50–90°C for 24 h (Fig 6d), all samples achieved effective consolidation, with compressive strengths exceeding 7 MPa and stabilizing above 50°C. This indicates that the polymerization and cross-linking reactions of MF resin complete at moderate temperatures, with limited further strength improvement at higher temperatures. Accelerated condensation between hydroxymethyl groups at elevated temperatures increases the cross-linking density, resulting in stable mechanical performance within the 60–90°C range, confirming the system’s suitability for sand consolidation in medium- to high-temperature reservoirs.

### 3.3. Performance of self-generated foam–resin composite sand consolidation system

#### 3.3.1. Foaming performance analysis.

The foaming properties of the optimized composite system were evaluated using the Waring Blender method. As shown in Table 6, the incorporation of resin-based sand consolidation components reduced the foam volume from 384 mL to 331 mL but significantly extended the foam half-life from 9 min to 14 min. This improvement is attributed to the weak acidity of the curing agent, which moderately suppressed foaming, combined with the viscosity enhancement provided by MF resins that contributed to foam stability. The composite system achieved a comprehensive foam value of 3475.5 mL·min^-1^, superior to the 2592 mL·min^-1^ of the foaming agent-alone system, demonstrating enhanced overall foam stability.

**Table 6:** Evaluation of foaming performance.

Composition	Foaming Volume/ mL	Half-Life/min	Comprehensive Foam Value/ mL·min^-1^
0.5% Foaming Agent	384	9	2592
Compound System	331	14	3475.5

#### 3.3.2. Consolidation performance and selective adhesion characteristics.

Sand pack experiments (Fig 7a, 7b) demonstrated that within the temperature range of 60–90°C, the cured cores exhibited compressive strengths exceeding 7.0 MPa while maintaining stable water permeability around 4 μm^2^, confirming the system’s ability to form a stable framework with both mechanical strength and flow capacity [43].

After thermal treatment, the system transitioned from liquid to viscous state, indicating partial resin polymerization. No adhesion was observed on clean steel surfaces, while a thin, water-rinsable cured film formed on sand-coated steel plates (Fig 7c). This selective adhesion characteristic confirms that the resin preferentially reacts with silanol and clay mineral groups on sand surfaces without forming stable bonds with metal substrates. The 60-minute erosion test provides an effective method for evaluating short-term stability under dynamic fluid impact [44].

#### 3.3.3. Erosion resistance and sand production rate.

Dynamic water flooding experiments (50 mL/min, 60 min) (Table 7) showed that cured cores prepared with sands of different particle sizes all exhibited sand production rates below 0.1% with no visible sand production at the outlet. These results demonstrate the system’s reliable sand control capability under continuous fluid flushing conditions, providing important evidence for field applications.

**Table 7:** Experimental results of erosion resistance of consolidated cores filled with quartz sand of different mesh sizes.

Mesh	Flow Rate/ mL/min	Time/ min	Mass Before Flushing/g	Mass After Flushing/g	Sand Production Rate/%	Note
40-60	50	60	123.65	123.53	0.097	Without sand
60-80			129.17	129.08	0.069	Without sand
80-100			132.54	132.47	0.052	Without sand
100-120			133.62	133.55	0.052	Without sand

#### 3.3.4. Sand consolidation mechanism.

**Fig 8:**
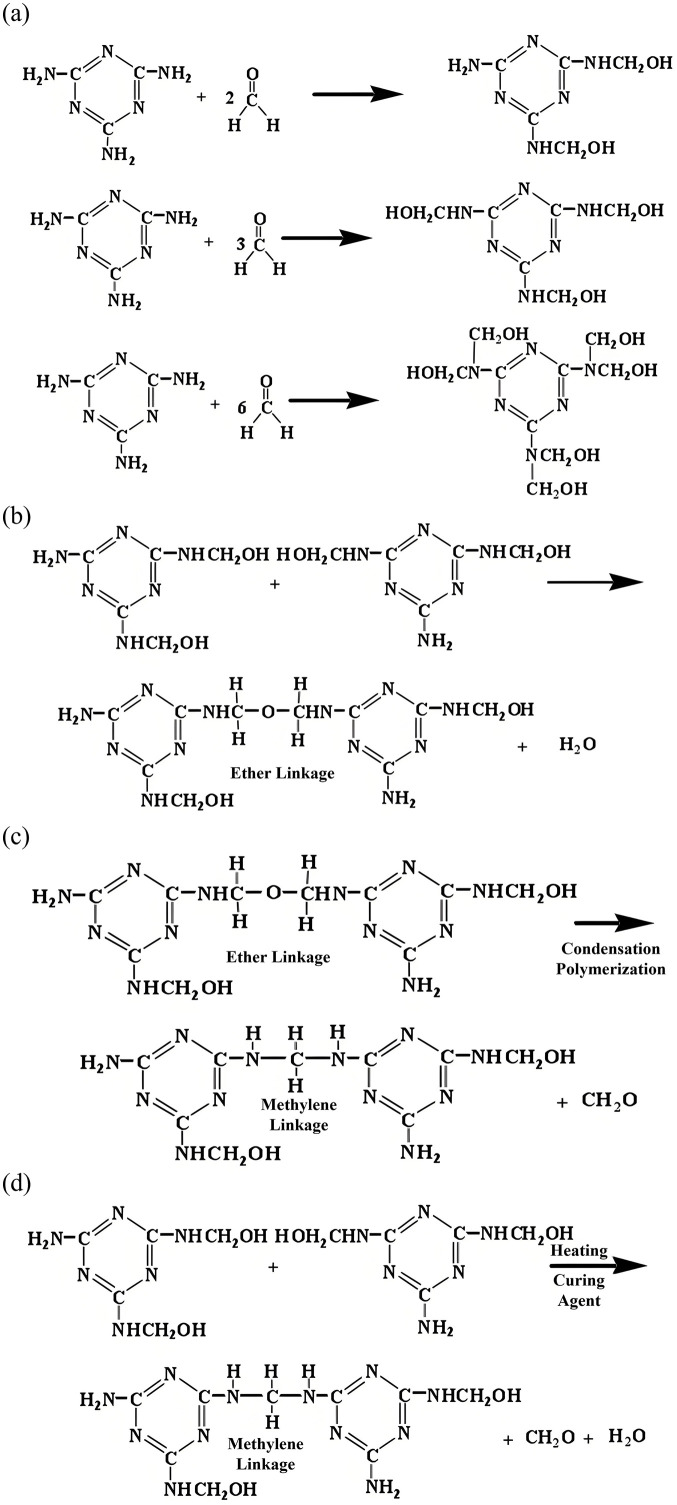
Reaction mechanism of the resin synthesis and curing process (a) chemical equation for the formation of hydroxymethyl prepolymer; (b) polymerization reaction process; (c) methylenation process; (d) chemical reaction process with the addition of curing agent.

The consolidation mechanism of the self-generated foam–resin composite system represents a sophisticated interplay between chemical polymerization and interfacial engineering. The process initiates under alkaline conditions (pH 8–9) where melamine undergoes nucleophilic addition with formaldehyde, generating hydroxymethylated prepolymers as primary intermediates (Fig 8a) [45]. This hydroxymethylation stage is crucial as it determines the subsequent cross-linking density and ultimate mechanical properties of the cured resin.

The polymerization then progresses through polycondensation, where hydroxymethyl (–CH₂OH) groups on adjacent melamine molecules undergo dehydration reactions. This stage primarily yields ether linkages (–CH₂–O–CH₂–) that form the backbone of the three-dimensional polymer network (Fig 8b). The curing process undergoes significant transformation during thermal treatment, where the initially formed ether bonds demonstrate limited thermal stability. These bonds undergo partial cleavage and rearrangement into more robust methylene bridges (–CH₂–) through a process termed methylenation (Fig 8c) [46]. This structural evolution from ether to methylene linkages represents a critical enhancement in the network’s thermal and mechanical stability.

The curing agent (NH₄Cl) plays a multifaceted role in this process. Beyond merely accelerating the reaction kinetics, it actively promotes the direct formation of methylene linkages while suppressing undesirable side reactions (Fig 8d). The ammonium ions catalyze the condensation reactions while the chloride ions help maintain the appropriate ionic strength for controlled polymerization. This coordinated action results in a densely cross-linked resin matrix with enhanced mechanical integrity and chemical resistance.

**Fig 9:**
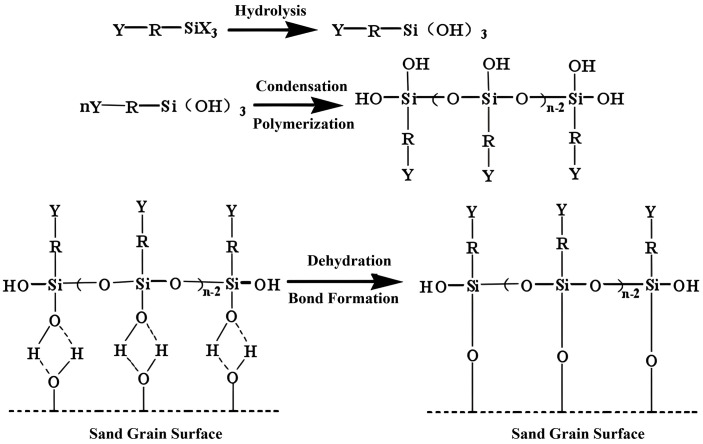
Bridging effect of silane coupling agent.

Simultaneously, the silane coupling agent KH-550 establishes vital connectivity between the organic resin and inorganic sand substrates. The hydrolyzed silanol groups of KH-550 condense with surface hydroxyls on quartz sand, forming strong covalent Si–O–Si bonds. Concurrently, the organofunctional groups of the coupling agent copolymerize with the developing resin network, creating a seamless organic-inorganic interface (Fig 9). This “molecular bridge” effect ensures efficient stress transfer between the resin matrix and sand grains, significantly enhancing the interfacial shear strength.

However, the interfacial optimization demonstrates a concentration-dependent behavior. Excessive KH-550 leads to multilayer adsorption and self-condensation, resulting in a weak boundary layer that compromises the interfacial integrity. This phenomenon explains the previously observed optimal concentration of 0.25% and underscores the importance of precise formulation control.

The exceptional performance of the consolidated sand packs—demonstrating compressive strengths exceeding 7 MPa, maintained permeability around 4 μm², and negligible sand production (<0.1%)—stems from the hierarchical integration of these mechanisms. The covalent cross-linking within the resin matrix provides the primary mechanical strength, while the silane-mediated interfacial bonding ensures efficient load transfer to the sand skeleton. The curing agent optimization balances reaction kinetics with network perfection, and the controlled foaming behavior enables uniform distribution of the consolidating agent throughout the sand pack.

This multi-scale understanding of the consolidation mechanism, spanning from molecular-level chemical transformations to macroscopic mechanical performance, provides a scientific foundation for optimizing sand control systems for challenging reservoir conditions. The demonstrated synergy between chemical polymerization and interfacial engineering offers valuable insights for developing next-generation sand consolidation technologies.

## 4. Conclusions

(1) Based on the comprehensive research, this study has successfully developed a self-generated foam–resin composite sand consolidation system, providing an efficient and reliable sand control solution for offshore heavy oil reservoirs. Through an optimized formulation (12.5% NaNO₂, 15% NH₄Cl, 2.5% catalyst, 0.5% foaming agent 20204, 25% melamine-formaldehyde resin, 1% curing agent, 0.25% coupling agent KH-550), the system achieves exceptional performance with consolidation strength >7 MPa and permeability ~4 μm^2^, maintaining stability within the 60–90°C temperature range. The system’s unique selective adhesion characteristic ensures preferential consolidation of formation sand without wellbore equipment plugging, while its long-term reliability is further demonstrated by a sand production rate of <0.1% in dynamic erosion tests.
(2) The sand consolidation mechanism relies on the synergistic effect between melamine-formaldehyde resin polymerization cross-linking and KH-550 interfacial bridging: under alkaline conditions, hydroxymethyl prepolymers form and construct a three-dimensional network through polycondensation; during thermal curing, ether bonds transform into more stable methylene bridges, while the silane coupling agent establishes a “molecular bridge” between the resin and sand grains to enhance interfacial bonding. This multi-scale synergistic mechanism enables the system to simultaneously achieve high strength, appropriate permeability, and exceptional erosion resistance.
(3) This research not only presents a technically viable sand consolidation method but also establishes a theoretical foundation for more environmentally friendly and sustainable offshore oil and gas development. Future work will focus on field trials and techno-economic assessments to promote the large-scale application of this technology, offering a new paradigm for intelligent sand control in complex reservoirs.
